# *Schistosoma japonicum* Associated With Colorectal Cancer

**DOI:** 10.14309/crj.0000000000000572

**Published:** 2021-05-11

**Authors:** Anas Almoghrabi, Obaie Mzaik, Bashar Attar

**Affiliations:** 1 Department of Gastroenterology, John H Stroger Jr. Hospital of Cook County, Chicago, IL; 2 Faculty of Medicine, University of Damascus, Damascus, Syria

## Abstract

Colorectal cancer (CRC) is one of the most common cancers worldwide, with increasing prevalence in Asian countries with a crude incidence of 21.1 per 100,000. *Schistosoma* is a genus of trematodes that infect millions of humans, affecting multiple organs, notably the intestines, liver, and bladder. Those trematodes may cause chronic inflammation in the affected organ leading to long-term complications such as fibrosis and neoplasia. There is rising evidence that infection with *Schistosoma japonicum* is correlated with the liver and CRC in endemic Asian countries. It is reported that chronic infection with Schistosomiasis raises the risk of CRC by 3 times. Less commonly seen outside of endemic areas, we present a case of *S. japonicum*-associated CRC in the United States in a woman with sigmoid adenocarcinoma and *Schistosoma japonicum* infection.

## INTRODUCTION

The global burden of colorectal cancer (CRC) has been rising rapidly with population growth, changes in demographics, and the westernization of lifestyle habits. CRC is the third commonest diagnosed cancer and the second leading cause of cancer-related mortality.^1^ Approximately, 70% of CRC cases are sporadic, likely influenced by environmental factors.^2^ Schistosomiasis, caused by blood-dwelling flukes, is one of the most prevalent parasitic diseases. Five schistosome species are known to cause human infection: *Schistosoma haematobium*, *Schistosoma mansoni*, *Schistosoma mekongi*, *Schistosoma intercalatum*, *and Schistosoma japonicum*. Among these species, *S. japonicum* is considered the most virulent because of the larger number of eggs it can produce as compared to other species, causing severe disease pathology.^3^ In addition, the zoonotic nature of *S. japonicum* contributes to increased disease transmission, making schistosomiasis control difficult.^3^ There is rising evidence that infection with *S. japonicum* is correlated with CRC in endemic Asian countries.^4^

## CASE REPORT

A 67-year-old woman from the Philippines with a medical history of psoriatic arthritis, hypertension, osteoporosis, and resected left-sided invasive ductal carcinoma of the breast presented with complaints of chronic lower abdominal discomfort associated with constipation alternating with diarrhea for a year, along with decreased appetite and 20 lbs. weight loss. She denied fever, bright red blood per rectum, or melena. She migrated to the United States from the Philippines in 1998. She never had a colonoscopy in the past. Her family history was negative for cancer. Vital signs and physical examination were unremarkable. Laboratory tests were noncontributory other than normocytic anemia with hemoglobin of 11.6 g per deciliter, no eosinophilia.

**Figure 1.**
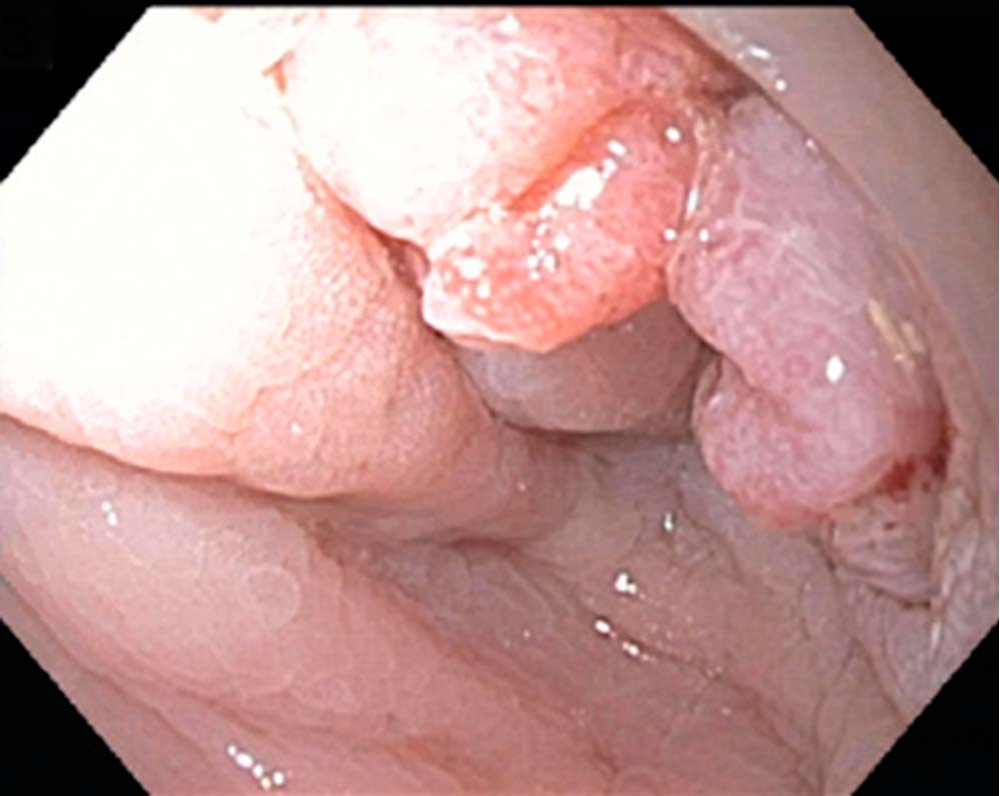
Colon mass in the sigmoid 20 cm from the entry site.

**Figure 2.**
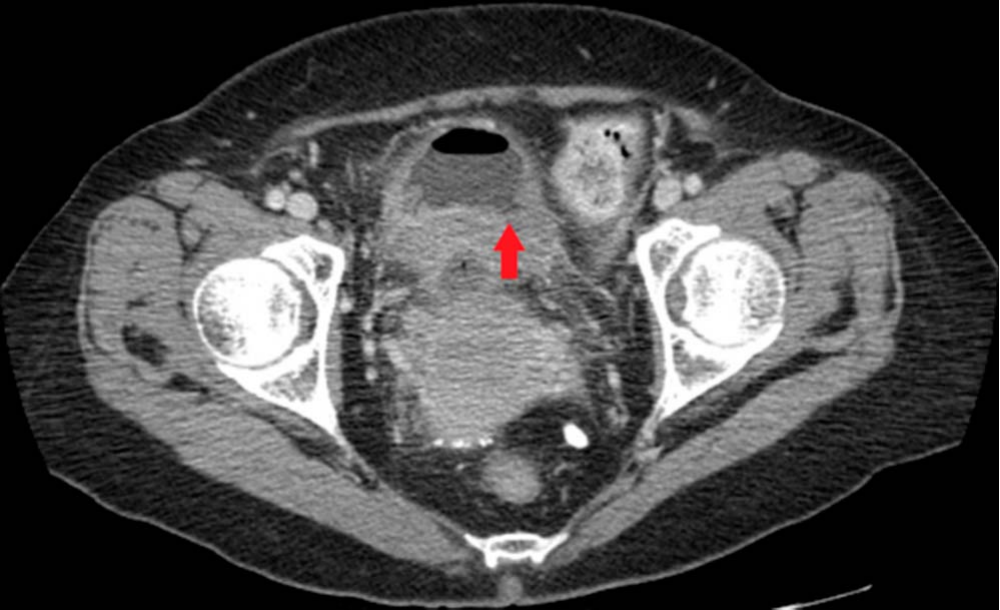
Computed tomography showing bladder wall thickening (red arrow).

**Figure 3.**
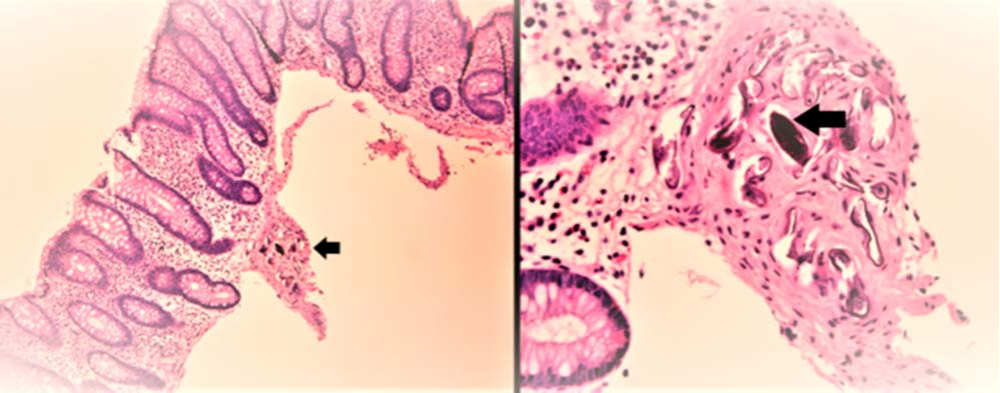
*Schistosoma japonicum* eggs (black arrows)—surgical pathology from the resected sigmoid.

Subsequent colonoscopy revealed a malignant appearing obstructing mass in the distal sigmoid, around 20 cm from the entry site (Figure 1). Biopsies from the mass revealed fragments of adenocarcinoma. Pelvic computed tomography (CT) revealed bladder wall thickening (Figure 2). Thoracic and abdominal CT revealed no distant metastasis. She underwent successful anterior pelvic exenteration, sigmoid resection with primary anastomosis, and ileal conduit creation for sigmoid cancer invading the bladder. Surgical pathology showed invasive, moderately differentiated adenocarcinoma in the sigmoid colon and numerous Schistosoma eggs in the colon, ovaries, posterior cul de sac, and pelvic wall (Figure 3). Along with surgical treatment, the patient was treated with praziquantel (60 mg/kg/d in 2 divided doses). Stool microscopic examination after 4 weeks of treatment was negative for ova and parasites.

## DISCUSSION

Schistosomiasis is an endemic disease in tropic and subtropic regions that infects a few 100 million people worldwide.^5^ Clinical manifestations may present acutely or chronically. The onset of symptoms depends on the stage of infection and ranges from local dermatitis, at the site of Schistosoma entry to Katayama fever, a systemic hypersensitivity response that presents with fever, fatigue, urticaria, and/or hepatosplenomegaly. Chronic manifestations include hepatic, intestinal, and urinary Schistosomiasis.^6^ Colonic Schistosomiasis is most caused by *S. mansoni* and *S. japonicum*. A study that evaluated 216 patients with schistosomal colonic disease showed that the most common presentation is nonspecific abdominal pain or distention (39% of study cases). Diarrhea, constipation, or blood in stool can be present. Other less reported complications include intestinal perforation, small bowel obstruction, colonic intususseption, ischemic colitis, colonic polyposis, and malignancy.^6–12^ *S. japonicum*-associated CRC has unique characteristics, including young age at diagnosis, male predominance, distal colonic location, multifocal distribution, and poor prognosis.^4^

Schistosoma infestation has been implicated by the International Agency for Research on Cancer as an etiology for several cancers such as colon, liver, and bladder. *S. japonicum* is classified as a probable carcinogen in humans (class 2B). Patients with chronic schistosomiasis japonica have 3 times the risk of developing colon cancer than those with no previous exposure to schistosomal infection.^4,13^ In a report by the National Cooperative Group on Pathology and Prognosis of CRC, the 5-year survival rate was 45.6% of 430 cases complicated with Schistosomiasis, which was significantly lower than in those without Schistosomiasis (50.9% of 2,717).^14^

Chronic inflammation caused by *S. japonicum* may be a promoting factor or a direct carcinogenic factor in adenocarcinoma development. Schistosoma provokes a chronic inflammation that activates inflammatory cells such as macrophages leading to the generation of reactive oxygen species and cytokines, both are genotoxic mediators that are proposed to cause DNA damage, mutations, and dysregulation of oncogenes and tumor suppressor genes.^4^ Tumor suppressor gene p53 mutations were found in a study of 22 Chinese patients with schistosomal rectal cancer.^15^ Furthermore, schistosomal eggs may induce colonic crypt elongation, hypertrophy, and a high percentage of atypical hyperplasia leading to epithelial proliferative type colonic polyposis.^16^

Our patient is from the Philippines, where 28 of 80 provinces are believed to be endemic with *S. japonicum*, and CRC incidence in 2018 was 15.2 per 100,000 among women and 23.5 per 100,000 among men.^17,18^ She migrated to the United States in 1998. Although the worms that cause Schistosomiasis are not found in the United States, people can be affected in other regions before coming to the United States. In the United States, it is estimated that at least 400,000 individuals are infected. Most of these are immigrants.^19^ We believe that chronic *S. japonicum* infection likely is the etiology of colon cancer in our patient. In western countries, physicians should be aware of such associations, especially for immigrants from endemic areas, to facilitate early diagnosis and management. The causal relationship and benefit of screening and treatment for Schistosomiasis are debated and need further research. Control of *S. japonicum* in endemic areas may decrease CRC incidence.^4^

## DISCLOSURES

Author contributions: A. Almoghrabi wrote the article and is the article guarantor. O. Mzaik reviewed the literature. B. Attar edited the article and revised the article for intellectual content.

Financial disclosure: None to report.

Previous presentation: This case was presented at the American College of Gastroenterology Annual Scientific Meeting; October 5-10, 2018; Philadelphia, Pennsylvania.

Informed consent was obtained for this case report.
